# Histamine2-Receptor Antagonists, Proton Pump Inhibitors, or Potassium-Competitive Acid Blockers Preventing Delayed Bleeding After Endoscopic Submucosal Dissection: A Meta-Analysis

**DOI:** 10.3389/fphar.2019.01055

**Published:** 2019-09-19

**Authors:** Xin Jiang, Jiahao Li, Jingmei Xie, Zhuoru Liang, Ning Wan, Jie Jiang, Tiantian Zhang, Yingyu Wu

**Affiliations:** ^1^School of International Pharmaceutical Business, China Pharmaceutical University, Nanjing, China; ^2^College of Pharmacy, Jinan University, Guangzhou, China; ^3^International Cooperative Laboratory of Traditional Chinese Medicine Modernization and Innovative Drug Development of Chinese Ministry of Education (MOE), Jinan University, Guangzhou, China; ^4^Department of Pharmacy, Guangzhou General Hospital of Guangzhou Military Command, Guangzhou, China; ^5^Guangzhou Huabo Biopharmaceutical Research Institute, Guangzhou, China; ^6^Dongguan Institute of Jinan University, Guangzhou, China

**Keywords:** endoscopic submucosal dissection, proton pump inhibitors, histamine_2_-receptor antagonists, vonoprazan, delayed bleeding

## Abstract

**Background:** Endoscopic submucosal dissection (ESD) was commonly used for en bloc resection in gastric cancer and adenoma with the risk of delayed bleeding after ESD. We conducted a direct and indirect comparison meta-analysis to evaluate the best choice in preventing post-ESD bleeding among proton pump inhibitors (PPIs), histamine_2_-receptor antagonists (H_2_RAs), and the most widely used potassium-competitive acid blocker, vonoprazan.

**Methods:** The Pubmed, Cochrane Library, and Embase were searched for randomized trials. We pooled odds ratios (OR) for preventing post-ESD bleeding using meta-analysis.

**Results:** Sixteen randomized trials met the inclusion criteria including 2,062 patients. Direct comparisons showed slightly significant efficacy in PPIs rather than H_2_RAs in preventing post-ESD bleeding [OR: 1.83; 95% confidence interval (CI): 1.10 to 3.05] and vonoprazan was better than PPIs (OR: 0.46; 95% CI: 0.25 to 0.86). The adjusted indirect comparison indicated vonoprazan was superior to H_2_RAs (OR: 0.30, 95% CI: 0.12 to 0.74). In subgroup analysis, PPIs had similar efficacy as H_2_RAs in 4 weeks, while PPIs were better than H_2_RAs in 8 weeks’ treatment (OR: 1.91; 95% CI: 1.08 to 3.40). The superiority of vonoprazan than PPIs was more significant in combination therapy (OR: 0.18; 95% CI: 0.04 to 0.69). There was a significant difference in vonoprazan for 8 weeks of medication (OR: 0.44; 95% CI: 0.21 to 0.92).

**Conclusions:** The effects of vonoprazan is better than PPIs than H_2_RAs in preventing bleeding after ESD. When vonoprazan combined with mucosal protective antiulcer drug in treatment or used in 8 weeks of medication, the efficacy may be even better.

## Introduction

Endoscopic submucosal dissection (ESD), as an endoscopic procedure, commonly used to treat gastric cancer and adenoma, was first reported in Japan and Korea in the late 1990s (Lightdale, 2004; Fujishiro, 2006). ESD is performed using a variety of electrosurgical knives such as the insulated-tip or the triangle-tip models as to make gastrointestinal mucosal incisions and submucosal dissections (Miyamoto et al., 2002; Yamamoto et al., 2003; Miyashita et al., 2003). Unlike conventional techniques such as the strip biopsy or cap endoscopic mucosal resection (EMR), the en bloc resection can be achieved by ESD (Rosch et al., 2004). Nowadays, it has gained popularity in Asian countries. With technical improvements and structured training, ESD is increasingly used in Western countries, and it is necessary to master the operation of this technology and vigorously promote it (Fukami, 2014).

However, ESD, as a more complex and time-consuming procedure, has a higher risk of complications than classic EMR, mainly related to bleeding (Oka et al., 2006). Delayed bleeding occurs mostly within 24 h after ESD, and the incidence rate of delayed bleeding is approximately 5% (Takizawa et al., 2008). Although rare, it can develop severe complications such as delayed perforation, and blood will interfere with subsequent endoscopic procedures. Hence, prevention of bleeding after ESD has been an important clinical issue.

The bleeding was affected by pH levels, and the gastric pH may affect the efficiency of blood coagulation and platelet aggregation at the bleeding site (Green et al., 1978; Barer et al., 1983). To neutralize pH level and prevent bleeding after ESD, there are some alternative medication options that include proton pump inhibitors (PPIs), histamine_2_-receptor antagonists (H_2_RAs), and so on. PPIs have been reported to be preferred than H_2_RAs treatment in some previous studies (Daneshmend et al., 1992; Esaki et al., 2002; Abe et al., 2004; Ye et al., 2006). Conversely, other studies showed that PPIs are not significantly effective than H_2_RAs (Ohya et al., 2010; Imaeda et al., 2011; Jang et al., 2012; Tomita et al., 2012). Vonoprazan (Takeda Pharmaceutical co., Tokyo, Japan) is a new potassium-competitive acid blocker (P-CAB). Its pharmaceutical advantages over PPIs include more rapid, stronger, and long-lasting inhibition of gastric acid secretion after administration (Jenkins et al., 2015; Sakurai et al., 2015). Moreover, vonoprazan effect is neither affected by CYP2C19 polymorphisms in its pharmacokinetics (Kagami et al., 2016) nor affected by food intake (Sachs et al., 2000; Ozawa et al., 2003). As a new class of acid-suppressing agents, it is expected to reduce the incidence of delayed bleeding after ESD better than conventional PPIs and serve as an alternative to PPIs (Hori et al., 2011; Jenkins et al., 2015; Sakurai et al., 2015).

The relative efficacy of PPIs, H_2_RAs, and the P-CAB (vonoprazan) has been assessed in randomized trials. However, the most appropriate management in preventing post-ESD bleeding remains controversial and to be defined. Moreover, the consumption of PPIs is especially high nowadays, and the overuse of PPIs has been an international concern (Lanas, 2016). Hence, in our study, we performed a meta-analysis among H_2_RAs, PPIs, and vonoprazan to find out the best regimen to prevent bleeding after ESD. Despite the absence of head-to-head randomized control trials (RCTs), we conducted indirect comparison meta-analysis to estimate the efficacy of vonoprazan and H_2_RAs using PPI as the common comparator.

## Methods

### Data Sources and Searches

The manuscript was drafted by the preferred reporting items for systematic reviews and meta-analyses statement (Moher et al., 2009). We searched three electronic databases: Pubmed, Cochrane Library, and Embase, from their inception date to 15th March 2019. There are no restrictions on abstracts and conference proceedings. We hand searched the relevant articles’ references to extend the literature search. The following terms were used in the search, which were included in their titles, abstracts, or keyword lists: “endoscopic submucosal dissection,” “ESD,” “proton pump inhibitors,” “PPI,” “vonoprazan,” “Potassium-Competitive Acid Blocker,” “H2Ras.” Only English publications were included.

### Study Selection and Endpoints

The inclusion criteria used to select studies are predetermined. The eligible studies are as follows: 1) RCTs that compared the efficacy of H_2_RAs or vonoprazan against PPIs on prevention of bleeding after ESD, not during the operation procedure, 2) the study population are adults aging from 18 years old, 3) studies must report the data of delayed bleeding incidence after ESD, and 4) trials were excluded by not about the prevention of bleeding. We chose the delayed bleeding as the primary outcome. Two independent reviewers (Tiantian Zhang and Jiahao Li) retrieved all the potentially relevant articles. Any discrepancies between the two independent reviewers were resolved by a third investigator (Ning Wan).

### Data Extraction and Quality Assessment

The data were extracted by two reviewers (Tiantian Zhang and Jiahao Li) independently and included the name of the first author, year of publication, country, a total number of patients, age, sex of patients, therapeutic regimen, trial name, tumor location, tumor depths, tumor with scar, mean resected specimen, and histopathology. Each study was judged on the potential bias including low, high, and unclear as outlined in the Cochrane Collaboration Handbook (Higgins et al., 2011).

### Data Analysis

In the absence of trials making head to head comparisons between H_2_RAs and vonoprazan, we then performed an adjusted indirect comparison using a common comparator—that is, PPIs—as described by the method of Bucher (Glenny et al., 2005). When few direct studies are available and direct comparative studies have not been performed, indirect meta-analysis is used to estimate treatment effect and to strengthen the power of comparisons instead of replacing randomized trials of direct comparison (Collaborative overview of randomised trials of antiplatelet therapy–II, 1994; Song et al., 2000; Fisher et al., 2001; Packer et al., 2001). We performed traditional meta-analysis combining trials of H_2_RAs versus PPIs, vonoprazan versus PPIs, to obtain the estimated odds ratios (OR) with 95% confidence interval (CI). We calculated the I^2^ statistic and the Chi-square test to examine statistical heterogeneity among studies. If the heterogeneity results were considered to be statistically significant at a *P* value < 0.1 and an I^2^ statistic > 50%, the random-effects model would be much more appropriate. Otherwise, the fixed-effects model was used. To strengthen the reliability of these pooled results and explore possible reasons for heterogeneity, we performed a sensitivity analysis using the leave-one-out method and examined the publication bias by funnel plot and Egger’s test. All tests were two-sided, and a P < 0.05 was considered statistically significant (Higgins et al., 2003; Fox et al., 2012; Loke et al., 2014). We used the STATA statistical software system v14.0 for statistical analysis.

## Results

### Literature Search

**Figure 1:**
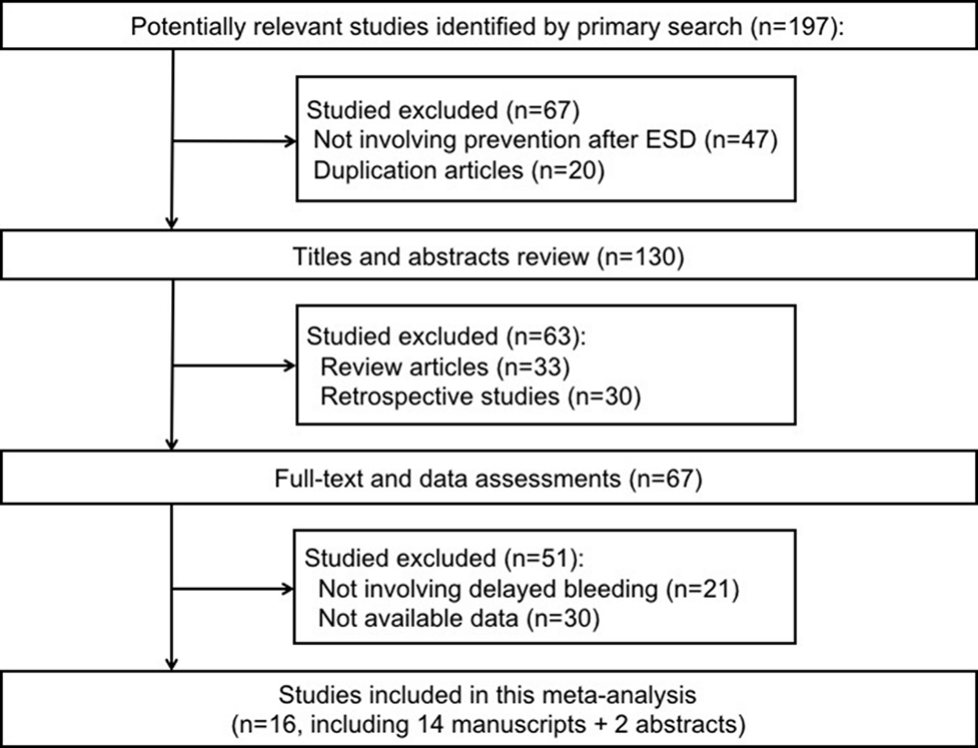
Preferred reporting items for systematic reviews and meta-analyses flowchart of the studies included in the meta-analysis.

The process of study selection in our meta-analysis is shown in **Figure 1**. Overall, the literature search identified 197 potentially relevant studies in our initial search. We excluded 130 articles for the following reasons: not involving prevention after ESD, review articles, duplicate articles, and retrospective studies. The remaining 67 articles were retrieved for further consideration. There were 51 articles that were excluded for not involving delayed bleeding and without sufficient data. Only 16 articles were included in the analysis including two conference abstracts (Jeong et al., 2007; Uedo et al., 2007; Ohya et al., 2010; Imaeda et al., 2011; Tomita et al., 2012; Jang et al., 2012; Kagawa et al., 2016; Ichida et al., 2018; Ishii et al., 2018; Hamada et al., 2018; Horikawa et al., 2018; Yamasaki et al., 2018).

### Study Characteristics and Qualities

A total of 1,956 patients were enrolled in the meta-analysis including 16 trials. The characteristic of the 16 included articles is shown in **Table 1**. The quality of the randomized studies is shown in **Table 2**.

**Table 1:** Clinical trials information.

Study	Trial name	Country	Year	Total number of patients	Regimen	No. of patients	Medication weeks	Age	Sex(male/female)	Tumor location (U/M/L)	Tumor location (body/antrum)	Tumor depths (m/sm)	Tumor with scar (+/-)	Mean resected specimen size/mm^2^+SD	Histopathology (adenoma/adenocarcinoma)
Noriya Uedo	NA	Japan	2007	143	PPIs	73	8	68.1 ± 8.5	57/16	NA	38/43	NA	12/69	41.0 ± 16.1	NA
					H2RAs	70	8	65.7 ± 7.6	55/15	NA	30/48	NA	9/69	40.5 ± 18.8	NA
Hye-Kyong Jeong	NA	Korea	2007	164	PPIs	85	8	62.9 ± 9.4	52/33	NA	26/59	NA	NA	31.1 ± 9.5	71/14
					H2RAs	79	8	63.5 ± 7.8	53/26	NA	22/57	NA	NA	31.1 ± 10	59/20
Tomohiko Richard Ohya	NA	Japan	2010	60	PPIs	31	4	65.4 ± 9.0	23/8	4/20/10	NA	33/1	NA	NA	NA
					H2RAs	29	4	65.3 ± 8.0	21/8	1/16/13	NA	27/3	NA	NA	NA
Hiroyuki Imaeda	UMIN000001069	Japan	2011	123	PPIs	62	8	68.4 ± 8.0	47/15	NA	42/20	59/2	12/50	NA	NA
					H2RAs	61	8	67.6 ± 8.5	52/9	NA	36/25	61/0	8/53	NA	NA
Toshihiko Tomita	UMIN000001215	Japan	2012	156	PPIs	77	8	70.4 ± 8.7	59/18	12/32/33	NA	66/11	7/70	43.8 ± 16.0	NA
					H2RAs	79	8	70.6 ± 9.5	59/20	16/33/30	NA	71/8	9/70	40.3 ± 15.6	NA
Jin Seok Jang ^†^	NA	Korea	2012	77	PPIs+Rebamipide	40	4	NA	NA	NA	NA	NA	NA	NA	NA
					H2RAs+Rebamipide	37	4	NA	NA	NA	NA	NA	NA	NA	NA
Myung H. Noh ^†^	NA	Korea	2013	190	PPIs+Cytoprotective Agents	92	4	NA	NA	NA	NA	NA	NA	NA	NA
					H2RAs+Cytoprotective Agents	98	4	NA	NA	NA	NA	NA	NA	NA	NA
T. Kagawa	UMIN000021010	Japan	2016	225	PPIs+ Polaprezinc	150	8	71.9 ± 9.1	91/59	20/33/97	NA	NA	NA	NA	26/124
					P-CAB (Vonoprazan)+ Polaprezinc	75	5	72.3 ± 8.4	52/23	11/12/52	NA	NA	NA	NA	10/65
Izumi Tsuchiya	UMIN000017077	Japan	2017	80	PPIs	41	8	74	30/11	5/15/19	NA	39/2	NA	NA	4/37
					P-CAB (Vonoprazan)	39	8	73	27/12	9/13/19	NA	39/0	NA	NA	2/37
Akira Yamasaki	NA	Japan	2017	167	PPIs	90	4	70 (42–90)	66/24	11/44/35	NA	NA	NA	NA	6/84
					P-CAB (Vonoprazan)	77	4	71 (39–87)	54/23	8/29/40	NA	NA	NA	NA	2/75
Yohei Horikawa	UMIN 000026391	Japan	2018	115	PPIs	53	2 weeks	73 (60–86)	34/19	15/20/18	NA	46/7	NA	NA	NA
					P-CAB (Vonoprazan)	62	2 weeks	69.5 (47–84)	44/18	12/24/26	NA	55/7	NA	NA	NA
Ai Hirai	UMIN000016687	Japan	2018	149	PPIs	75	8	69.9 ± 11.0	55/20	4/29/42	NA	71/4	NA	NA	7/68
					P-CAB (Vonoprazan)	74	8	73.2 ± 7.5	62/8	9/27/41	NA	60/14	NA	NA	8/66
Kenta Hamada	UMIN000017320	Japan	2018	139	PPIs	70	8	70.1 ± 8.5	57/23	NA	NA	NA	7/63	38 ± 15	NA
					P-CAB (Vonoprazan)	69	8	70.3 ± 6.8	51/18	NA	NA	NA	6/63	38 ± 14	NA
Yasuaki Ishii	MIN000016835	Japan	2018	53	PPIs	26	8	70 (66–75.3)	22/3	14/10/2	NA	NA	NA	39.8 (26-80)	NA
					P-CAB (Vonoprazan)	27	8	70 (65.3–75)	23/4	12/10/5	NA	NA	NA	40.6 (30-54)	NA
Hiroyuki Komori	UMIN000017386	Japan	2019	33	PPIs	15	4	70.9 ± 8.8	11/4	2/8/5	NA	NA	NA	NA	NA
					P-CAB (Vonoprazan)	18	4	69 ± 9.3	13/5	1/4/13	NA	NA	NA	NA	NA
Takashi Ichida	UMIN000019516	Japan	2019	82	PPIs+Rebamipide	39	8	73.9 (58–88)	34/5	4/18/17	NA	NA	NA	38.6 (21-66)	14/25
					P-CAB (Vonoprazan)+Rebamipide	43	8	72.4 (52–89)	31/12	7/12/24	NA	NA	NA	39.9 (18-66)	8/35

PPIs, proton pump inhibitors; H2RAs, histamine2-receptor antagonists; NA, not available; U/M/L, upper/middle/lower; m/sm, intramucosal cancer/submucosal invasive cancer; SD, standard deviation.

^†^abstract.

**Table 2:** Quality of the randomized studies.

Study (first author)	Random sequence generation	Allocation concealment	Blinding of participants and personnel	Blinding of outcome assessment	Incomplete outcome data	Selective reporting	Other sources of bias
Uedo et al., 2007	Low	Low	Low	Low	Low	Low	Unclear
Jeong et al., 2007	Low	Low	Low	Unclear	Unclear	Unclear	Unclear
Ohya et al., 2010	Low	Unclear	Unclear	Unclear	Unclear	Low	Unclear
Imaeda et al., 2011	Low	Low	Low	Low	Low	Low	Unclear
Tomita et al., 2012	Low	Unclear	Low	Low	Low	Low	Unclear
Jang et al., 2012 ^†^	Low	Unclear	Unclear	Unclear	Low	Unclear	Unclear
Noh et al., 2013	Low	Unclear	Unclear	Unclear	Low	Unclear	Unclear
Kagawa et al., 2016	Unclear	Unclear	Low	Low	Low	Unclear	Unclear
Yamasaki et al., 2018	Low	Unclear	Unclear	Low	Low	Low	Unclear
Tsuchiya et al., 2017	Low	Low	Low	Unclear	Unclear	Low	Unclear
Hirai et al., 2018	Low	Low	High	High	Unclear	Low	Unclear
Hamada et al., 2018	Low	Low	High	Low	Low	Unclear	Unclear
Ishii et al., 2018	Low	Low	High	Low	Low	Low	Unclear
Horikawa et al., 2018	Low	Unclear	Unclear	Low	Low	Low	Unclear
Komori et al., 2019	Low	Unclear	Unclear	Low	Low	Low	Unclear
Ichida et al., 2018	Low	Low	Low	Low	Low	Unclear	Unclear

^†^abstract.

### Conventional Meta-Analysis

**Figure 2:**
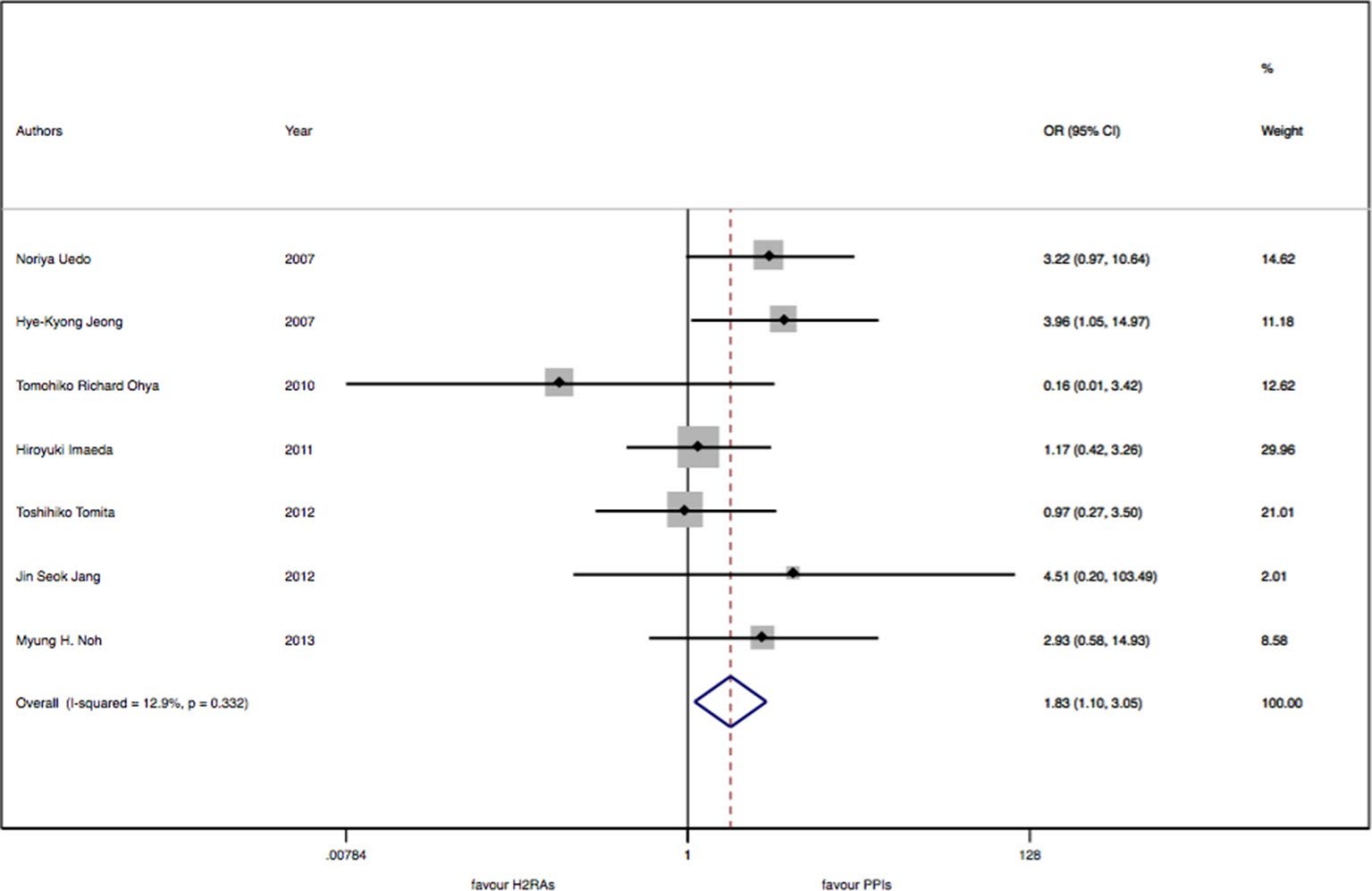
The meta-analysis of delayed bleeding for H_2_RAs with PPIs.

**Figure 3:**
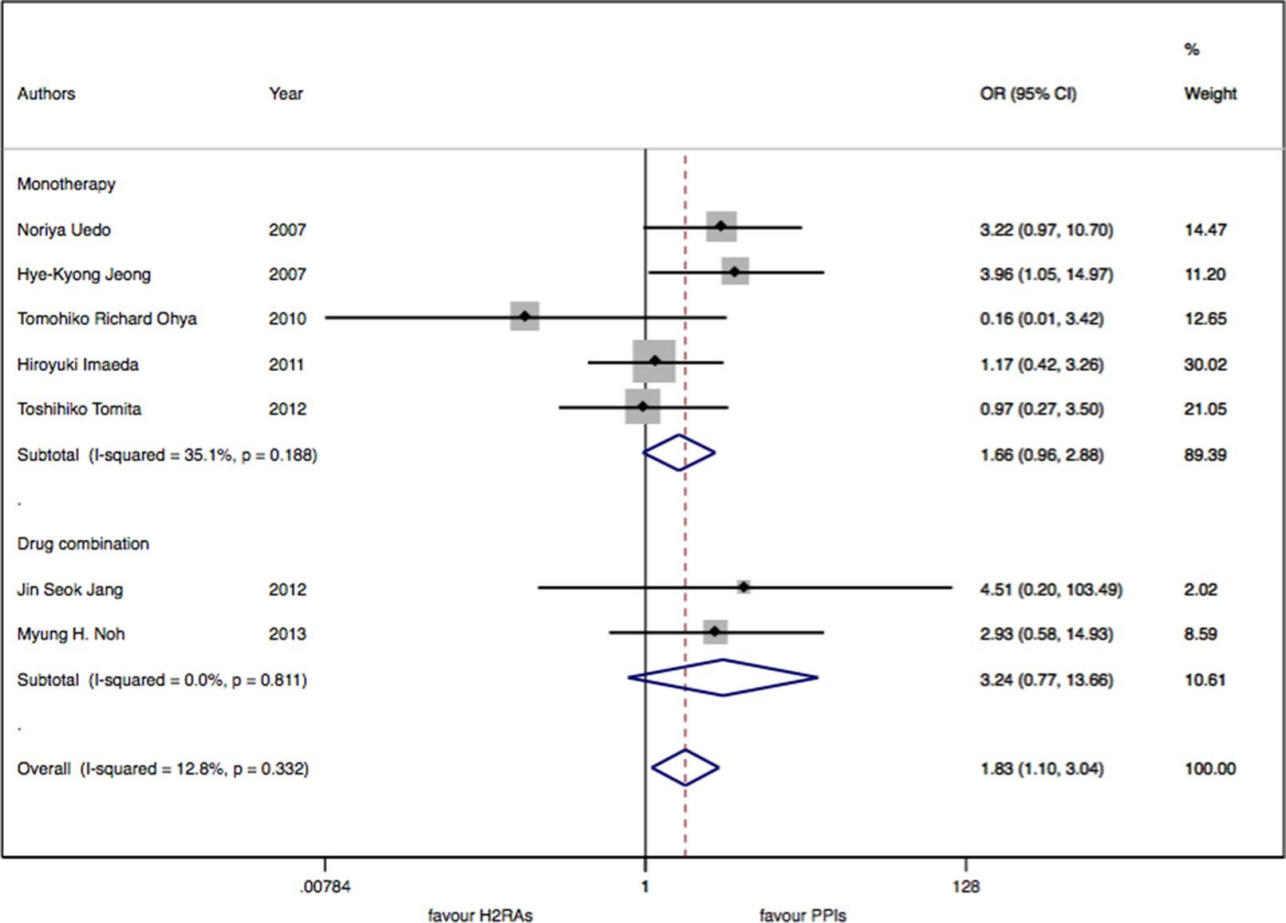
The medication duration subgroup meta-analysis of delayed bleeding for H_2_RAs with PPIs.

**Figure 4:**
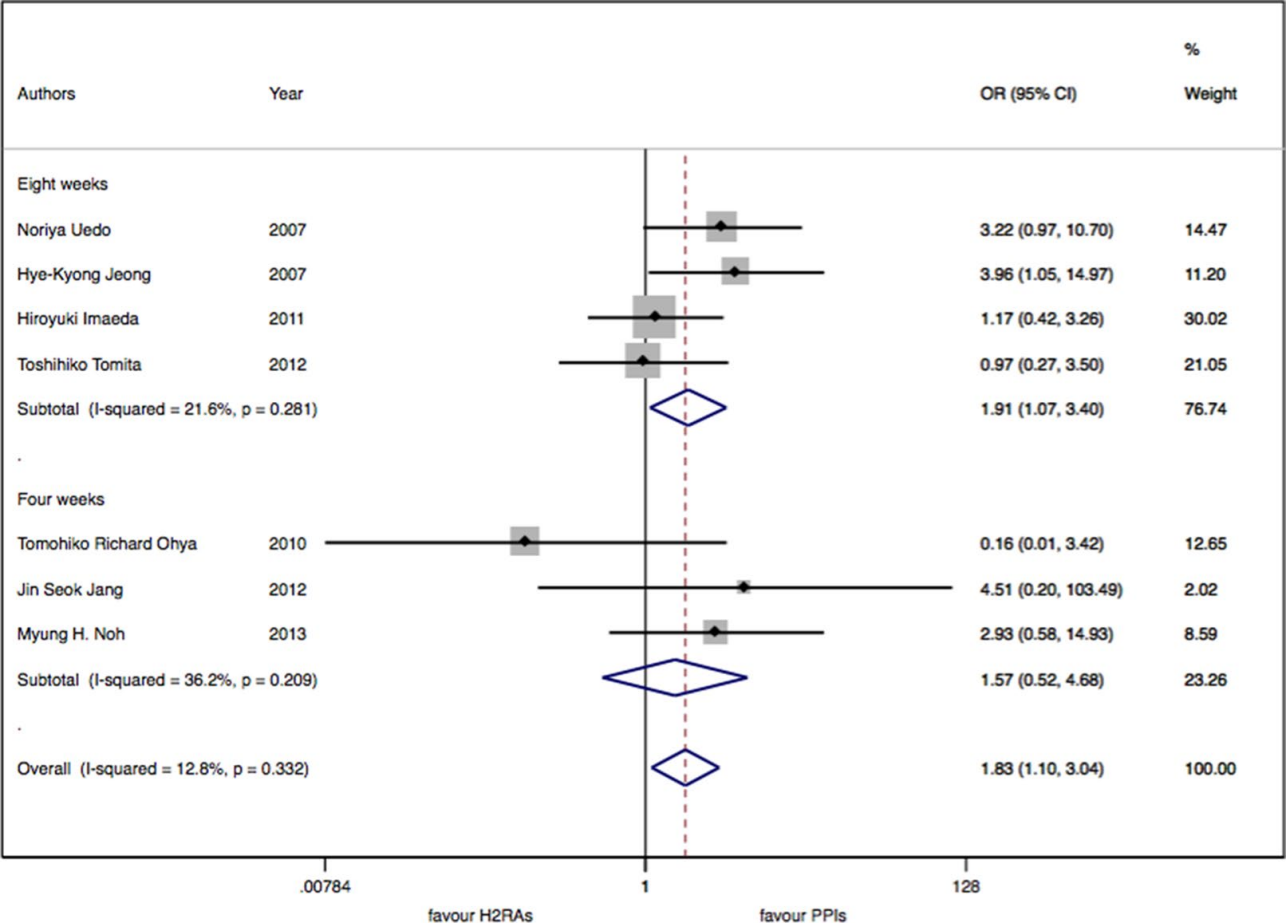
The medication regimen subgroup meta-analysis of delayed bleeding for H_2_RAs with PPIs.

There were seven studies with 913 patients about H_2_RAs versus PPIs. No significant statistical heterogeneity among trials was detected (I^2^ = 12.9%, P = 0.33). There was a slightly significant difference in delayed bleeding between the H_2_RAs and PPIs groups (OR: 1.83; 95% CI: 1.10 to 3.05, P = 0.02). However, this difference was not significant for 4 weeks (OR: 1.57; 95% CI: 0.52 to 4.68, P = 0.42). It was only for 8 weeks; PPIs were superior to H_2_RAs in delayed bleeding (OR: 1.91; 95% CI: 1.08 to 3.40, P = 0.028). Compared with the OR of PPIs monotherapy, the OR of H_2_RAs monotherapy was 1.66 (95% CI: 0.96 to 2.88). When combined with protective agents, the OR increased to 3.24 (95% CI: 0.77 to 13.66) (see **Figures 2**–**4**).

**Figure 5:**
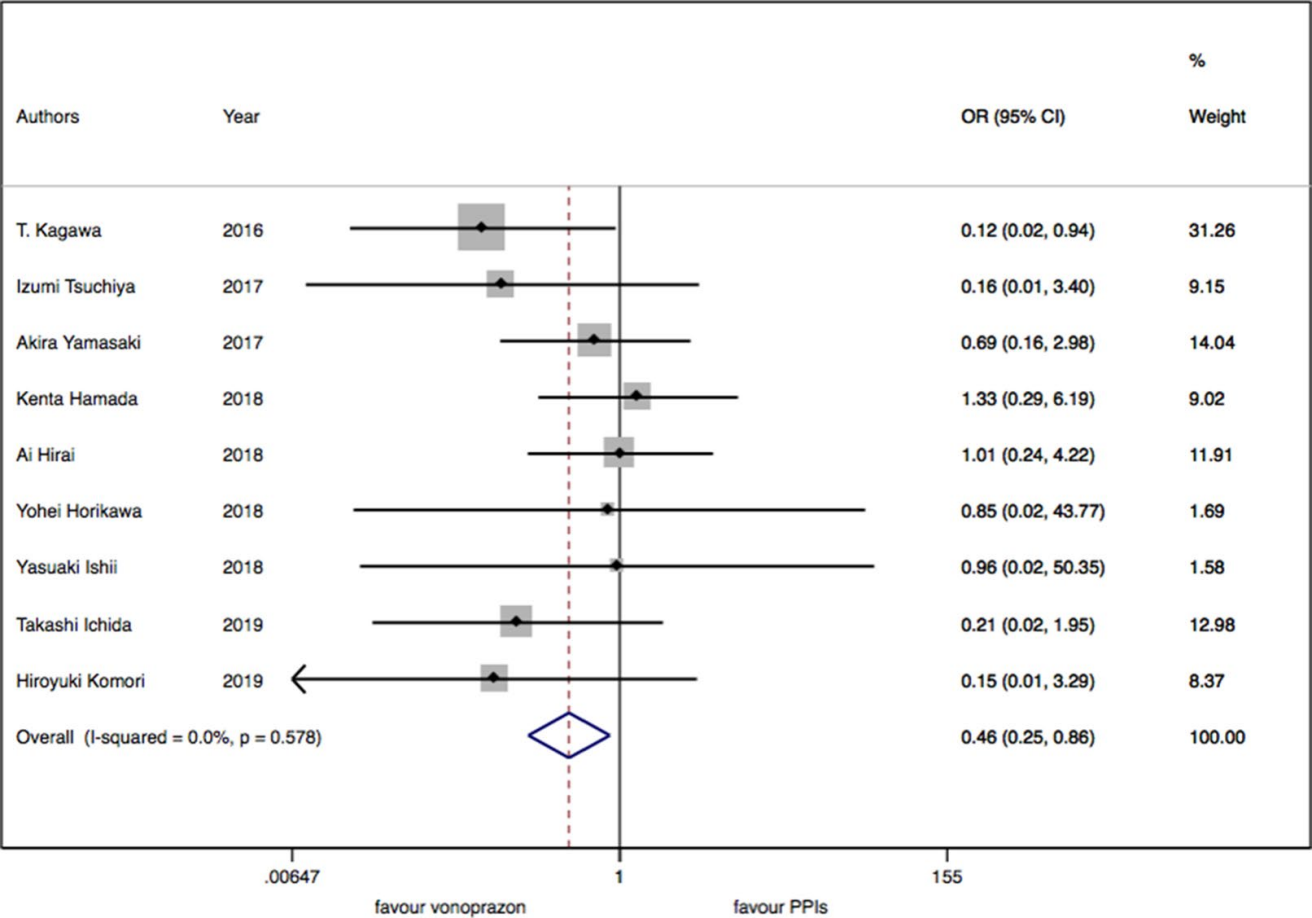
The meta-analysis of delayed bleeding for vonoprazan with PPIs.

**Figure 6:**
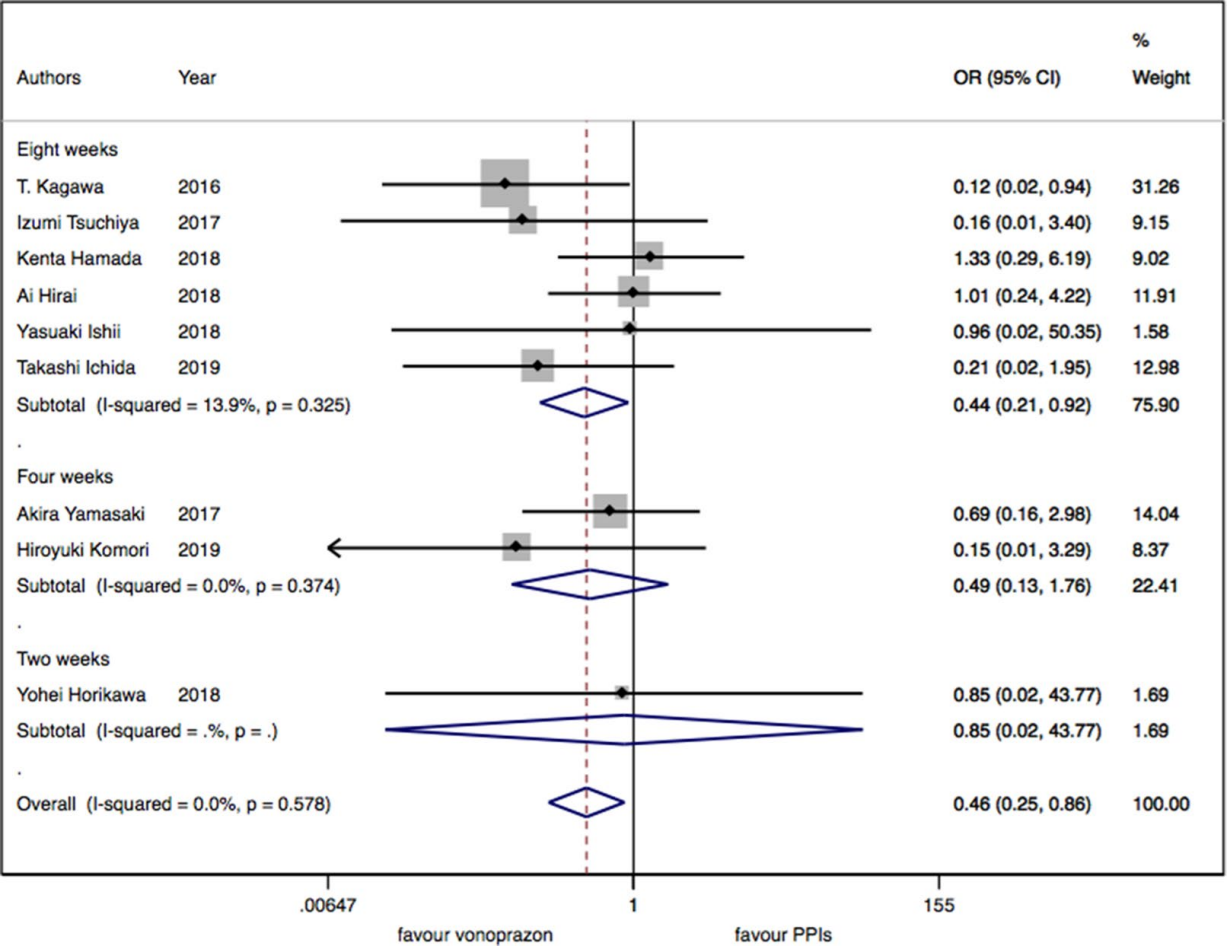
The medication duration subgroup meta-analysis of delayed bleeding for vonoprazan with PPIs.

**Figure 7:**
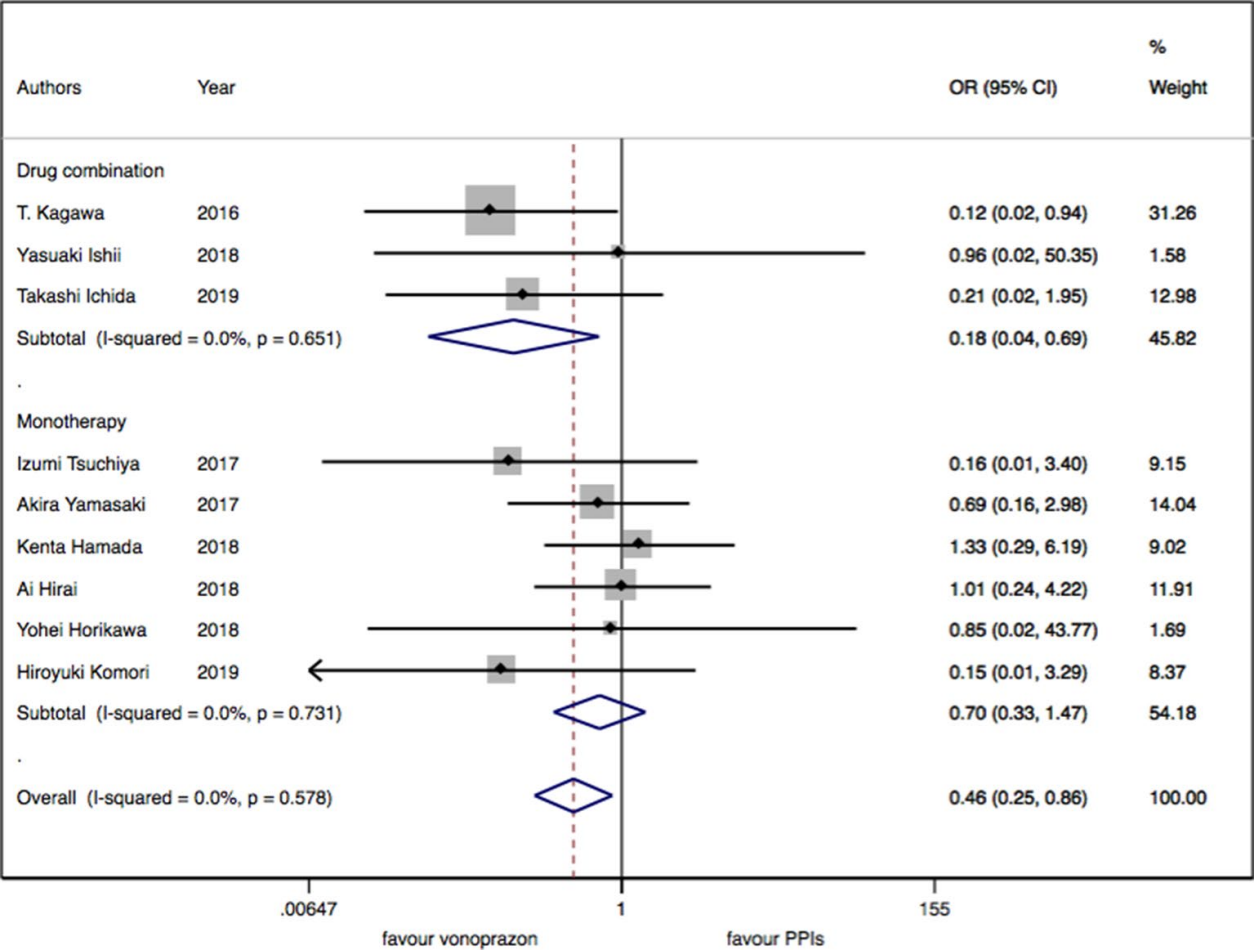
The medication regimen subgroup meta-analysis of delayed bleeding for vonoprazan with PPIs.

As for the nine trials of vonoprazan versus PPIs including 1,043 patients, a significant difference in delayed bleeding (OR: 0.46; 95% CI: 0.25 to 0.86, P = 0.015) was found in vonoprazan versus PPIs. In the subgroup analysis, there existed a significant difference in delayed bleeding when vonoprazan was combined with mucosal protective antiulcer drug in treatment compared with PPI combination therapy (OR: 0.18; 95% CI: 0.04 to 0.69, P = 0.013), while the OR was 0.70 for vonoprazan monotherapy (95% CI: 0.33 to 1.47). Additionally, vonoprazan showed better efficacy in delayed bleeding in 8 weeks’ medication than PPIs (OR: 0.44; 95% CI: 0.21 to 0.92) (see **Figures 5**–**7**).

### Adjusted Indirect Comparison Based on Common Controls

The adjusted indirect comparison of delayed bleeding was performed for vonoprazan and H_2_RAs using PPIs as the comparator. This demonstrated that vonoprazan was superior to H_2_RAs (OR: 0.30, 95% CI: 0.12 to 0.74). The same trend was found in the subgroup analysis of vonoprazan compared with H_2_RAs. The OR between vonoprazan and H_2_RAs of 8 weeks’ medication duration was 0.29 (95% CI: 0.10 to 0.86). The OR between vonoprazan and H_2_RAs in a combination regimen with protective agents was 0.061 (95% CI: 0.006 to 0.458).

### Sensitivity Analysis

**Figure 8:**
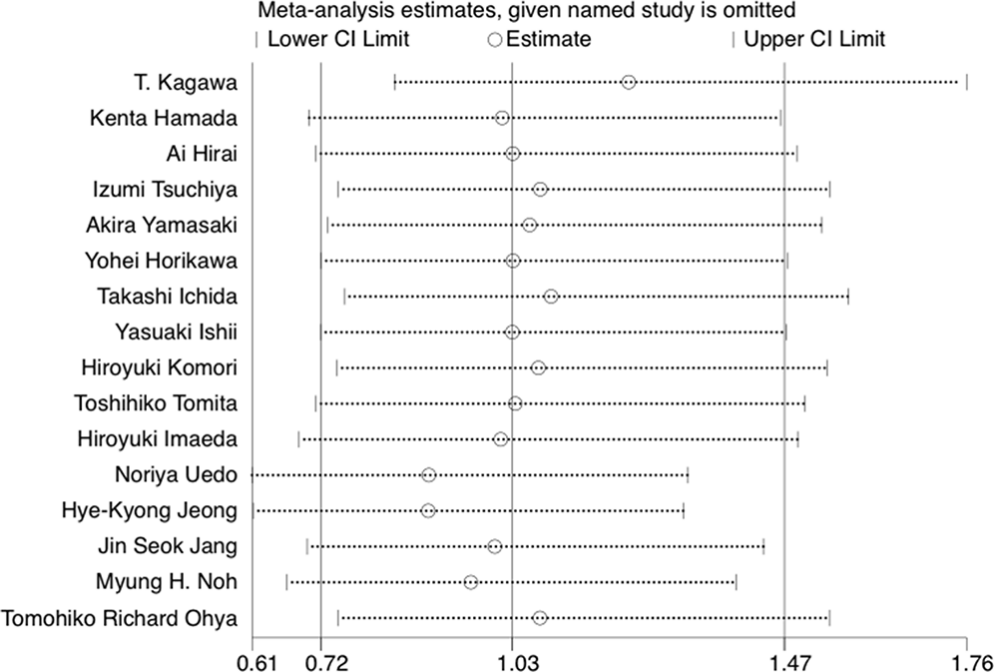
The leave-one-out sensitivity analysis of preventing bleeding after ESD per medication option.

**Figure 9:**
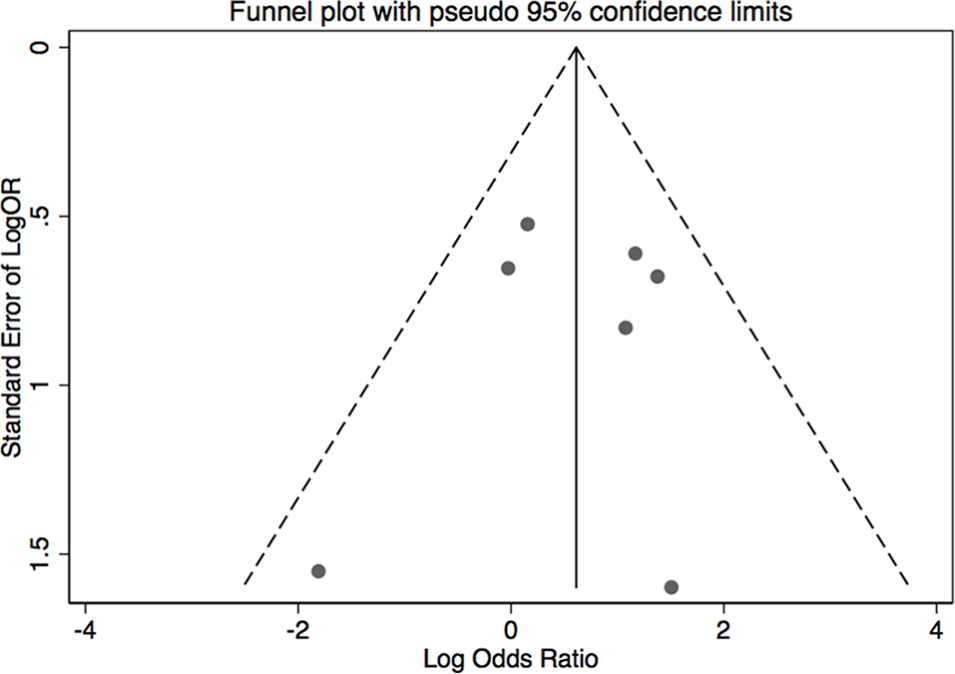
Funnel plot of the standard error of publication bias for H_2_RAs with PPIs.

**Figure 10:**
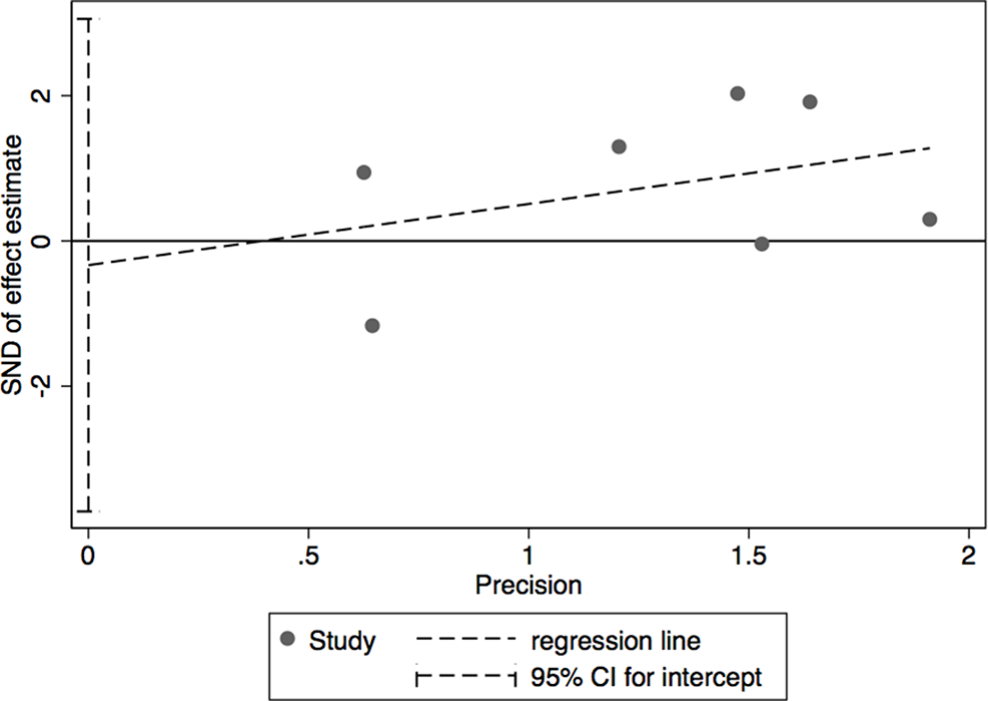
Egger’s plot of the standard error of publication bias for H_2_RAs with PPIs.

**Figure 11:**
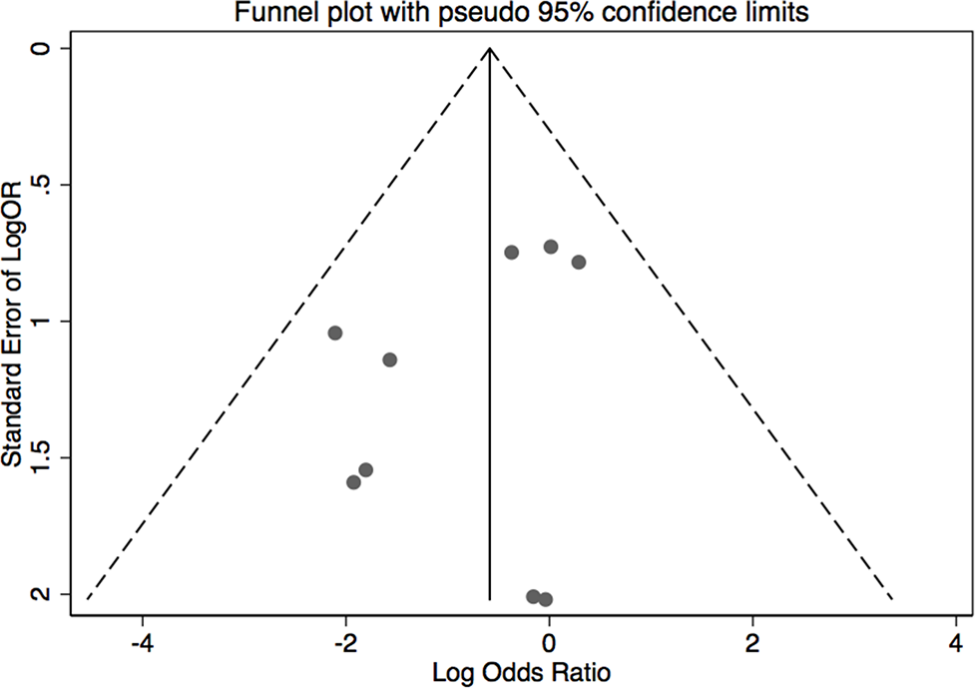
Funnel plot of the standard error of publication bias for vonoprazan with PPIs.

**Figure 12:**
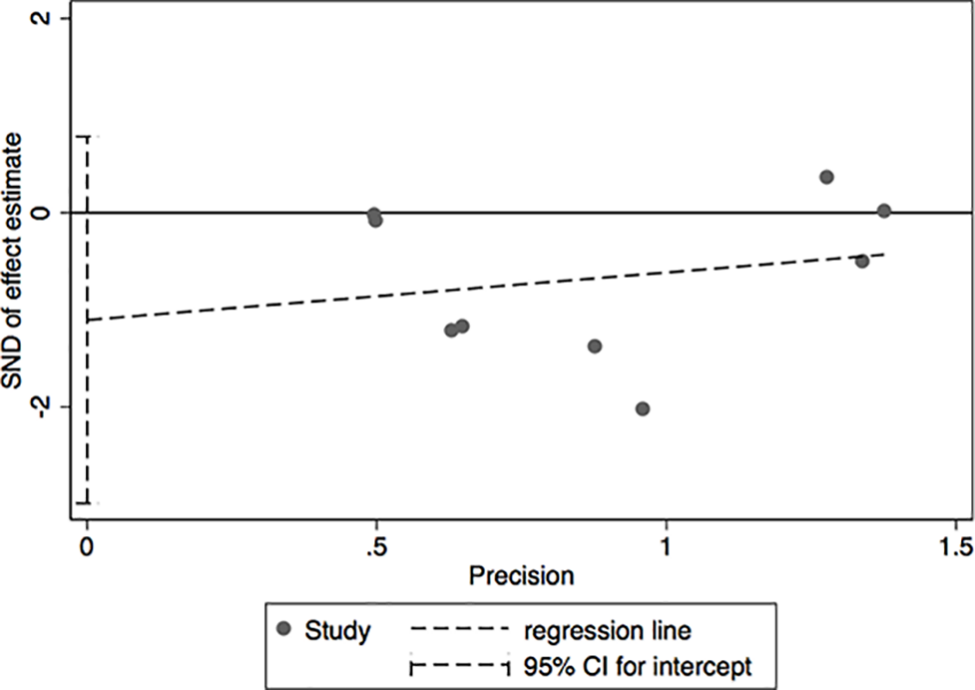
Egger’s plot of the standard error of publication bias for vonoprazan with PPIs.

The results of leave-one-out sensitivity analysis indicated that no individual studies significantly influenced the OR (see **Figure 8**). Based on the funnel plot and Egger’s test, no significant publication bias was found that may confirm the stability of our results (see **Figures 9**–**12**). The *P* values for Egger’s test were 0.810 for H_2_RAs with PPIs and 0.209 for vonoprazan with PPIs.

## Discussion

Based on the traditional meta-analysis, PPIs was inferior to vonoprazan in the prevention of bleeding after ESD, while it was superior to H_2_RAs. In the result of indirect comparison meta-analysis by using PPIs as an intermediary, there was a significant decrease in delayed bleeding in vonoprazan versus H_2_RAs. In the subgroup analysis, the superiority of PPIs than H_2_RAs was more obvious in 8 weeks of medication duration than 4 weeks. Meanwhile, the significant effect of vonoprazan versus PPIs was more obvious under the situation of combination therapy than monotherapy and 8 weeks medication duration than 4 weeks.

The rapid promotion of ESD calls for quality improvement and precise management in preventing post-ESD bleeding. To our knowledge, we represented the first step at finding out a more appropriate management system for delayed bleeding prevention including choice of drug, combination of medication, and medication duration by conducting both direct and indirect meta-analysis.

According to our research results, either vonoprazan or PPIs may have better efficacy in preventing post-ESD bleeding in 8 weeks of medication duration than 4 weeks. Additionally, vonoprazan–mucosal protective antiulcer drugs regimen was better than PPIs–mucosal protective antiulcer drugs. The probable explanation was that it may exist as a much stronger synergistic effect between the vonoprazan and mucosal protective antiulcer drugs than PPIs. Therefore, we recommended that the patients who have a high risk of bleeding including long operation time, large resection range, deeper tumor location, and the combination treatment with drugs of potential bleeding take vonoprazan–mucosal protective antiulcer drugs for 8 weeks. However, for patients under low risk of bleeding, shorter duration may be a better choice, taking both cost and adverse events into consideration.

In one previous meta-analysis (Yang et al., 2011), it indicated that PPIs were superior to H_2_RAs for the prevention of delayed bleeding. However, the author mixed the EMR and ESD studies to assess the delayed bleeding in the ESD subgroup analysis and may lead to some controversial results. Instead, our study focused on ESD specifically and compared the prevention efficacy of PPIs with H_2_RAs and vonoprazan for delaying the bleeding after ESD. In the medication duration of H_2_RA and PPI subgroup analysis, our results were consistent with the former study that revealed that PPIs exerted a better influence in 8 weeks’ medication duration than in 4 weeks. The reason may be that the actions of H_2_RAs are significantly faster than those of PPIs (Ahmad et al., 2002; van Rensburg et al., 2003). A recent meta-analysis compared the rate of healing of ulcers caused by ESD and post-ESD delayed bleeding between vonoprazan and PPIs (Jaruvongvanich et al., 2018). However, since three eligible studies were not included, the research concluded that no difference was found between vonoprazan and PPIs.

There were several limitations associated with our study. Firstly, we should not neglect the heterogeneity among different clinical trials. In our study, the regimens of the RCTs were diverse from each other. As the multivariate analysis indicated, the scar in the tumor as well as the size of the tumor, and the endoscopists’ skill, were independent predictors for bleeding (Uedo et al., 2007; Chung et al., 2009; Jang et al., 2009; Okada et al., 2011). All these factors may have an impact on clinical results. Further studies with a larger number of patients are required to clarify the association between the comorbidities and complications after ESD. Secondly, we could not analyze the safety of the treatments without enough adverse events data. Thirdly, there may exist a population bias in our study. All trials included in our research were implemented in Asia, and only one evidence was from Italy (Catalano et al., 2009). Therefore, the generalization of results should be taken in caution. On the other hand, the most appropriate drug administration time (before ESD or after ESD) and method (orally or intravenously) of PPIs still remained controversial. Meanwhile, even vonoprazan and PPI were preferable in efficacy; it may not be optimal choices in terms of their high cost compared with H_2_RAs. Therefore, more high-quality researches including cost-effectiveness analysis should be conducted to evaluate these issues and give some further guidance for clinical application.

In conclusion, our results show better effects of vonoprazan over PPIs over H_2_RAs in preventing bleeding after ESD. When vonoprazan was combined with mucosal protective antiulcer drug in treatment or used in 8 weeks of medication, the efficacy may be even better. So, as for the high-bleeding risk patients, it was recommended to take vonoprazan combination therapy or take vonoprazan for 8 weeks. However, due to the lack of adverse events data, more studies are needed on safety profiles of the previously discussed options and should be conducted on the Western population for the generalization of our results.

## Author Contributions

All authors were responsible for the structure of this paper. YW and TZ contributed to the conception and design. XJ and JL conducted the literature search and data analysis. JX, ZL, NW, and JJ checked for statistical inconsistency and interpreted data. XJ and JL drafted the paper. YW and TZ conducted critical revisions of the paper. All authors approved the final version for submission.

## Funding

This study was supported by the National Natural Science Foundation of China (grant no. 71704064), the Natural Science Foundation of Guangdong Province, China (grant no. 2017A030310174), and the Fundamental Research Funds for the Central Universities (grant no. 21616324).

## Conflict of Interest Statement

The authors declare that the research was conducted in the absence of any commercial or financial relationships that could be construed as a potential conflict of interest.
